# Supplementary material for: Ubiquitin Ligase Nrdp1 Controls Autophagy-Associated Acrosome Biogenesis and Mitochondrial Arrangement during Spermiogenesis
Source: Cells. 2023 Sep 5;12(18):2211. doi: 10.3390/cells12182211 (PMC10527437; doi:10.3390/cells12182211)
Supplement: Supplementary file 1 [file cells-12-02211-s001.zip › cells-2480079-Supplementary.pdf]

Supplementary Materials

Ubiquitin Ligase Nrdp1 Controls Autophagy-Associated Acrosome Biogenesis and Mitochondrial Arrangement during Spermiogenesis

Zi-Yu Luo [1,†], Tian-Xia Jiang [1,†], Tao Zhang [2], Ping Xu [2,*] and Xiao-Bo Qiu [1,*]

1 Ministry of Education Key Laboratory of Cell Proliferation & Regulation Biology, College of Life Sciences, Beijing Normal University, 19 Xinjiekouwai Avenue, Beijing 100875, China; 201111201017@mail.bnu.edu.cn (Z.-Y.L.); jiangtx@bnu.edu.cn (T.-X.J.)
2 State Key Laboratory of Proteomics, Beijing Proteome Research Center, Institute of Lifeomics, 38 Science Park Road, Beijing 102206, China; zhangtao120567648@163.com
* Correspondence: xuping@ncpsb.org.cn (P.X.); xqiu@bnu.edu.cn (X.-B.Q.)
† These authors contributed equally to this work.

Table S1. Proteins co-upregulated in Nrdp1$^{-/-}$sperm and testis haploid cells.

Gene names	protein description	Ratio in sperm (KO/Control)	Ratio in testis haploid cells (KO/Control)
Gpc4	Glypican-4; Secreted glypican-4	3.87	1.69
/	Ig kappa chain V-II region 26-10	2.38	3.46
Fkbp15	FK506-binding protein 15	2.31	1.51
Zfyve1	Zinc finger FYVE domain-containing protein 1	2.21	1.51
Emb	Embigin	2.01	2.80
Cldn3	Claudin-3	1.84	1.55
Nt5e	5-nucleotidase	1.72	1.99
Ctsh	Pro-cathepsin H	1.71	1.57
Spock2	Testican-2	1.68	1.65
Fam89b	Protein FAM89B	1.68	1.51
Gnaq	Guanine nucleotide-binding protein G(q) subunit alpha	1.65	1.66
Krt76	Keratin, type II cytoskeletal 2 oral	1.65	1.59
Pomgnt2	Protein O-linked-mannose beta-1,4-N-acetylglucosaminyltransferase 2	1.64	2.53
Atp6v0a1	V-type proton ATPase 116 kDa subunit a isoform 1	1.59	1.75
Slc16a1	Monocarboxylate transporter 1	1.57	1.78
Tmem106b	Transmembrane protein 106B	1.55	1.60
Lcp1	Plastin-2	1.54	1.67

KO: Nrdp1$^{-/-}$; Control: Nrdp1$^{+/-}$.

Table S2. Proteins upregulated in Nrdp1$^{-/-}$ testis haploid cells, but downregulated in Nrdp1$^{-/-}$ sperm.

Gene names	protein description	Ratio in sperm (KO/Control)	Ratio in testis haploid cells (KO/Control)
Fam96b	Mitotic spindle-associated MMXD complex subunit MIP18	0.38	2.19
Tmem263	Transmembrane protein 263	0.66	1.54
Cyp2b10	Cytochrome P450 2B10	0.53	1.69

KO: Nrdp1$^{-/-}$; Control: Nrdp1$^{+/-}$.

Table S3. Mitochondrium or lysosome-related proteins upregulated in Nrdp1$^{-/-}$ testis haploid cells.

Functions classification	Gene names	Protein descriptions	Ratio(KO/Control）
Mitochondria	Hadh	Hydroxyacyl-coenzyme A dehydrogenase, mitochondrial	2.5
	Hmgcs2	Hydroxymethylglutaryl-CoA synthase, mitochondrial	2.48
	Bcat2	Branched-chain-amino-acid aminotransferase, mitochondrial	2.47
	Txndc5	Thioredoxin domain-containing protein 5	2.41
	Hoga1	4-hydroxy-2-oxoglutarate aldolase, mitochondrial	2.4
	Aldh2	Aldehyde dehydrogenase, mitochondrial	2.19
	Slc25a4	ADP/ATP translocase 1	2.18
	Slc25a18	Mitochondrial glutamate carrier 2	2.11
	Prdx3	Thioredoxin-dependent peroxide reductase, mitochondrial	2.05
	Slc25a5	ADP/ATP translocase 2; ADP/ATP translocase 2, N-terminally processed	2.01
	Acads	Short-chain specific acyl-CoA dehydrogenase, mitochondrial	1.97
	Cds2	Phosphatidate cytidylyltransferase 2	1.96
	Idh2	Isocitrate dehydrogenase [NADP], mitochondrial	1.94
	Ak3	GTP: AMP phosphotransferase AK3, mitochondrial	1.91
	Aldh7a1	Alpha-aminoadipic semialdehyde dehydrogenase	1.88
	Gcdh	Glutaryl-CoA dehydrogenase, mitochondrial	1.87
Autophagy and lysosome	Plekhg5	Pleckstrin homology domain-containing family G member 5	2.82
	Scarb2	Lysosome membrane protein 2	2.66
	Ralb	Ras-related protein Ral-B	2.31
	Hk2	Hexokinase-2	1.85
	Vapb	Vesicle-associated membrane protein-associated protein B	1.85
	Vdac1	Voltage-dependent anion-selective channel protein 1	1.79
	Rptor	Regulatory-associated protein of mTOR	1.76
	Ppt1	Palmitoyl-protein thioesterase 1	1,75
	Atp6v0a1	V-type proton ATPase 116 kDa subunit a isoform 1	1.75
	Coro1a	Coronin-1A	1.64
	Tmem106b	Transmembrane protein 106B	1.6
	Gba	Glucosylceramidase	1.6
	Mapk3	Mitogen-activated protein kinase 3	1.58
	Pycard	Apoptosis-associated speck-like protein containing a CARD	1.57
	Myo7a	Unconventional myosin-VIIa	1.52
	Hdac6	Histone deacetylase 6	1.5

KO: Nrdp1$^{-/-}$; Control: Nrdp1$^{+/-.}$

Table S4. Mitochondrium or lysosome-related proteins upregulated in Nrdp1$^{-/-}$ sperm.

Functions classifications	Gene names	Protein descriptions	Ratio (KO/Control)
Mitochondria	Ndufv3	NADH dehydrogenase [ubiquinone] flavoprotein 3, mitochondrial	2.21
	Atp5d	ATP synthase subunit delta, mitochondrial	1.96
	Atp5a1	ATP synthase subunit alpha, mitochondrial	1.94
	Atp5h	ATP synthase subunit d, mitochondrial	1.91
	Atp5c1	ATP synthase subunit gamma, mitochondrial	1.83
	Ndufs6	NADH dehydrogenase [ubiquinone] iron-sulfur protein 6, mitochondrial	1.77
	Ndufb11	NADH dehydrogenase [ubiquinone] 1 beta subcomplex subunit 11, mitochondrial	1.76
	Ndufs4	NADH dehydrogenase [ubiquinone] iron-sulfur protein 4, mitochondrial	1.76
	Atp5f1	ATP synthase F(0) complex subunit B1, mitochondrial	1.74
	Atp5o	ATP synthase subunit O, mitochondrial	1.74
	Ndufb5	NADH dehydrogenase [ubiquinone] 1 beta subcomplex subunit 5, mitochondrial	1.73
	Atp5i	ATP synthase subunit e, mitochondrial	1.72
	Ndufb4	NADH dehydrogenase [ubiquinone] 1 beta subcomplex subunit 4	1.72
	Atp5e	ATP synthase subunit epsilon, mitochondrial	1.71
	Ndufa9	NADH dehydrogenase [ubiquinone] 1 alpha subcomplex subunit 9, mitochondrial	1.71
	Ndufa10	NADH dehydrogenase [ubiquinone] 1 alpha subcomplex subunit 10, mitochondrial	1.7
	Ndufb8	NADH dehydrogenase [ubiquinone] 1 beta subcomplex subunit 8, mitochondrial	1.7
	Ndufb9	NADH dehydrogenase [ubiquinone] 1 beta subcomplex subunit 9	1.68
	Ndufs8	NADH dehydrogenase [ubiquinone] iron-sulfur protein 8, mitochondrial	1.68
	Ndufa8	NADH dehydrogenase [ubiquinone] 1 alpha subcomplex subunit 8	1.67
	Ndufv1	NADH dehydrogenase [ubiquinone] flavoprotein 1, mitochondrial	1.67
	Ndufs1	NADH-ubiquinone oxidoreductase 75 kDa subunit, mitochondrial	1.66
	Ndufs3	NADH dehydrogenase [ubiquinone] iron-sulfur protein 3, mitochondrial	1.66
	Ndufa5	NADH dehydrogenase [ubiquinone] 1 alpha subcomplex subunit 5	1.63
	Ndufb10	NADH dehydrogenase [ubiquinone] 1 beta subcomplex subunit 10	1.63
	Ndufs7	NADH dehydrogenase [ubiquinone] iron-sulfur protein 7, mitochondrial	1.63
	Atp5b	ATP synthase subunit beta, mitochondrial	1.62
	Ndufs2	NADH dehydrogenase [ubiquinone] iron-sulfur protein 2, mitochondrial	1.6
	Ndufa2	NADH dehydrogenase [ubiquinone] 1 alpha subcomplex subunit 2	1.59
	Ndufs5	NADH dehydrogenase [ubiquinone] iron-sulfur protein 5;	1.59
	Ndufa3	NADH dehydrogenase [ubiquinone] 1 alpha subcomplex subunit 3	1.57
	Ndufv2	NADH dehydrogenase [ubiquinone] flavoprotein 2, mitochondrial	1.57
	Atp5j2	ATP synthase subunit f, mitochondrial	1.53
	Ndufa13	NADH dehydrogenase [ubiquinone] 1 alpha subcomplex subunit 13	1.53
	Ndufa6	NADH dehydrogenase [ubiquinone] 1 alpha subcomplex subunit 6	1.5

Autophagy and lysosome	Parkin (Park2)	E3 ubiquitin-protein ligase Parkin	1.69
	LAMP1	Lysosome-associated membrane glycoprotein 1	1.66

KO: Nrdp1$^{-/-}$; Control: Nrdp1$^{+/-}$.

Table S5. Acrosome-related proteins downregulated in Nrdp1$^{-/-}$ testis haploid cells.

Functions classification	Gene names	Protein descriptions	Ratio (KO/Control)
Germ cell differentiation	Hmgb2*	High mobility group protein B2	0.55
Acrosome biogenesis	Sirt1*	NAD-dependent protein deacetylase sirtuin-1	0.65
Manchette development	Meig1*	Meiosis-expressed gene 1 protein	0.66
	Rimbp3*	RIMS-binding protein 3	0.67

KO: Nrdp1$^{-/-}$; Control: Nrdp1$^{+/-}$. * Mutation or deletion of these proteins will lead to sperm defect in mice.

Table S6. Acrosome-related proteins downregulated in Nrdp1$^{-/-}$ sperm.

Functions classification	Gene names	Protein descriptions	Ratio (KO/Control)
Acrosome biogenesis	Zpbp2*	Zona pellucida-binding protein 2	0.35
	Acrbp*	Acrosin-binding protein	0.48
Golgi function	Spink2*	Serine protease inhibitor Kazal-type 2	0.42
Acrosome reaction	Cd46*	Membrane cofactor protein	0.42
	Cmtm2b*	CKLF-like MARVEL transmembrane domain-containing protein 2B	0.48
	Plcz1*	1-phosphatidylinositol 4,5-bisphosphate phosphodiesterase zeta-1	0.49
Acrosomal ingredient	Zp3r	Zona pellucida sperm-binding protein 3 receptor	0.34
	Acr	Acrosin;Acrosin light chain;Acrosin heavy chain	0.40
	Acrv1	Acrosomal protein SP-10	0.41
	Spaca3	Sperm acrosome membrane-associated protein 3	0.60

KO: Nrdp1$^{-/-}$; Control: Nrdp1$^{+/-}$.

* Mutation or deletion of these proteins will lead to sperm defect in mice.

Table S7. Expression of partial Nrdp1 substrates in Nrdp1$^{-/-}$ sperm.

Gene names	Protein descriptions	Ratio (KO/Control)
ErbB3	Epidermal growth factor (EGE) receptor 3	Undetectable
Dvl1[45]	Segment polarity protein dishevelled homolog DVL-1	1.03
BIRC6	Baculoviral IAP repeat-containing protein 6	0.96
Tbk1[46]	Serine/threonine-protein kinase TBK1	0.90
Dvl2[45]	Segment polarity protein dishevelled homolog DVL-2	0.79
Parkin (Park2)	E3 ubiquitin-protein ligase Parkin	1.69

KO: Nrdp1$^{-/-}$; Control: Nrdp1$^{+/-}$.

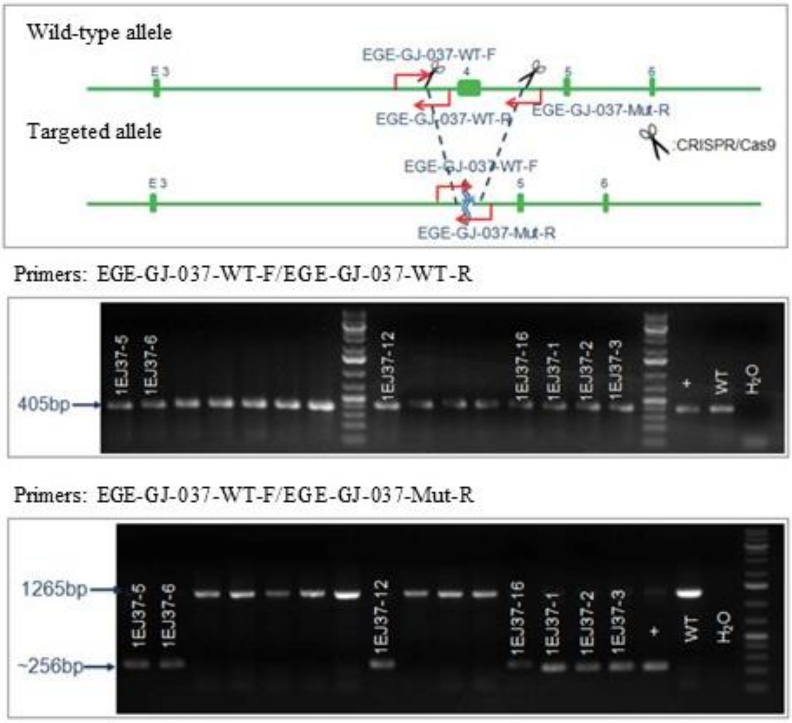

Figure S1. Crispr-Cas9 schematic diagram of *Nrdp1* deletion in mice. Nrdp1-deficient mouse genotype scheme. Product size: ca. 405 bp from the wild-type allele, ca. 230 bp from the knockout allele. PCR products were separated by agarose gel electrophoresis. 1EJ37-1~1EJ37-16, mouse number; +, positive control; WT, wild-type mice.

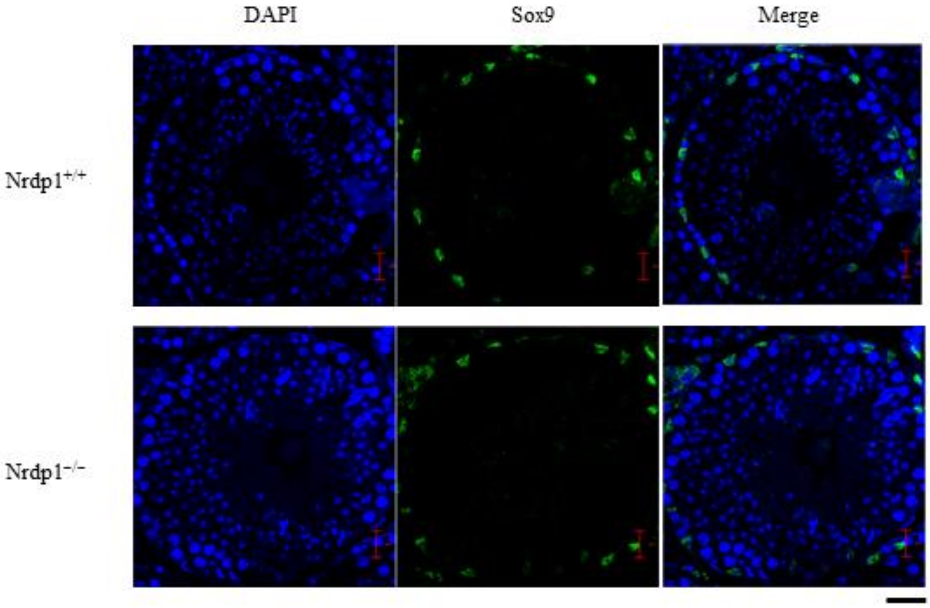

Figure S2. No difference in Nrdp1-deficient Sertoli cells is detectable. Testis sections were analyzed by immunofluorescence staining using antibody against Sox9 (green). The nuclei were stained by DAPI (blue). Scale bar=10 μm. Data are from two independent biological replicates of the same experiment.

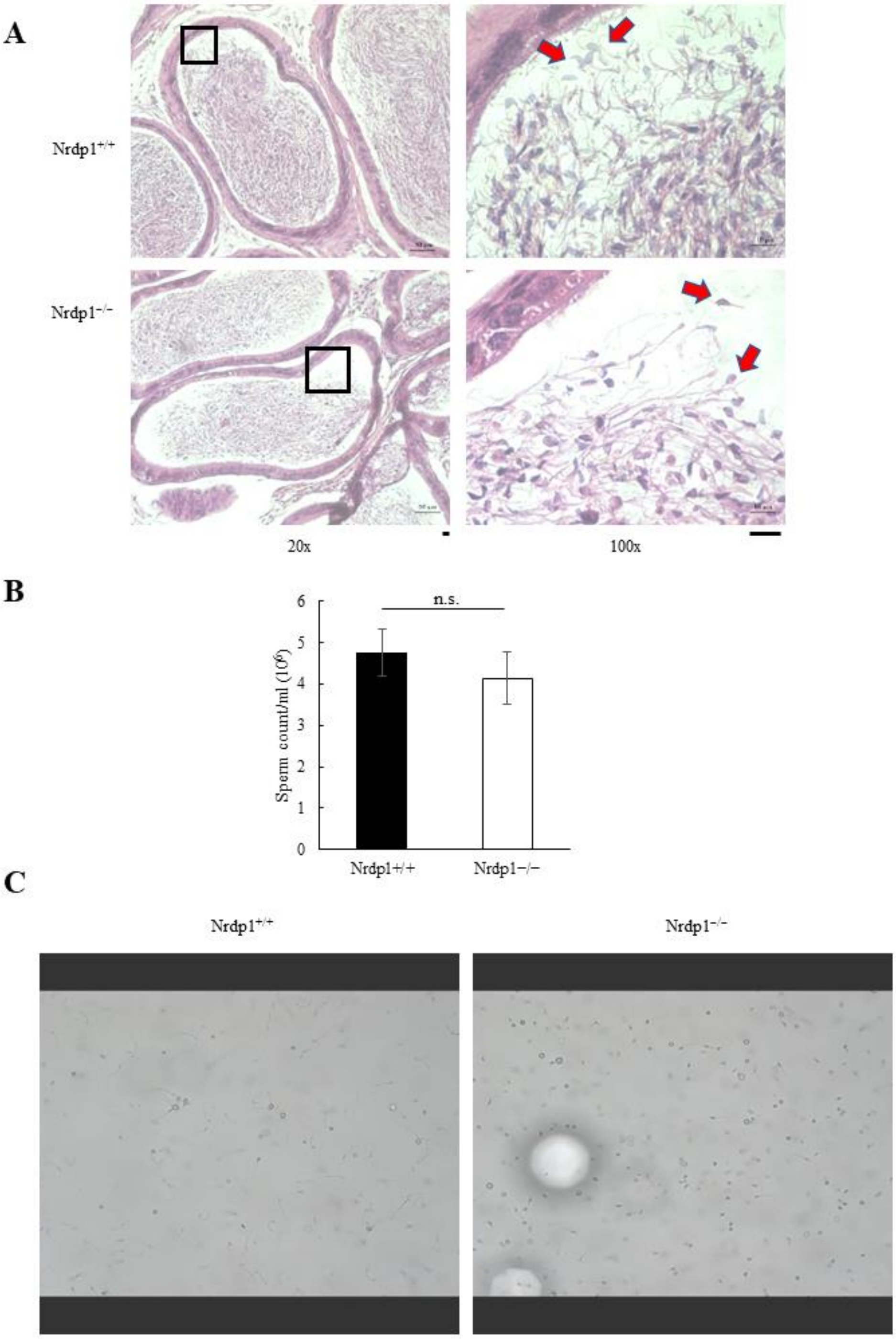

Figure S3. Motility of *Nrdp1*$^{-/-}$ sperm is defective. (A) Sections of the epididymis from *Nrdp1*$^{+/+}$ and *Nrdp1*$^{-/-}$ mice were examined by H&E staining. Red arrow indicates sperm. Scale bar= 10 µm. (B) Sperm were released from the cauda epididymis, and sperm number (×10^6 cells/ml) was counted. n=4. (C) Movie reflects sperm motility from *Nrdp1*$^{+/+}$ and *Nrdp1*$^{-/-}$ mice. Data are presented as mean ± SEM. n.s. stands for not significant. Data are from four independent biological replicates of the same experiment (two-tailed unpaired t-test in B).

A

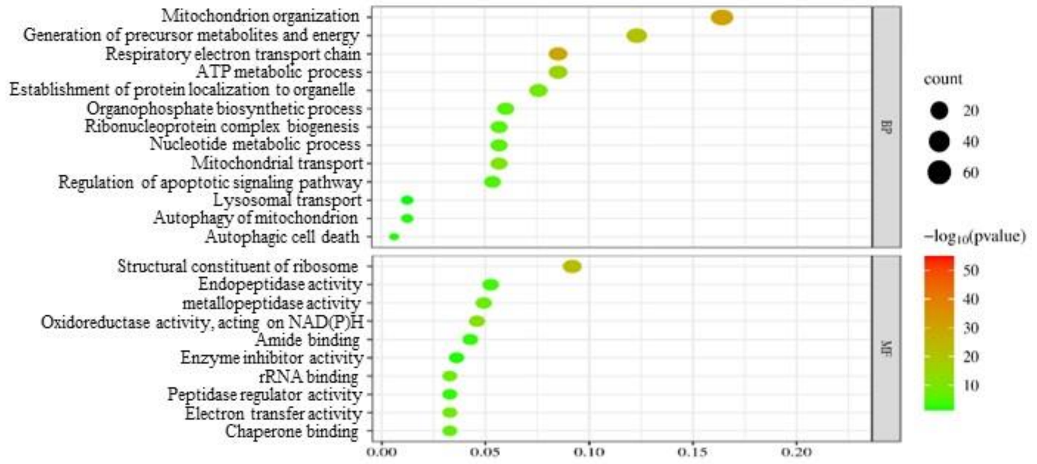

B

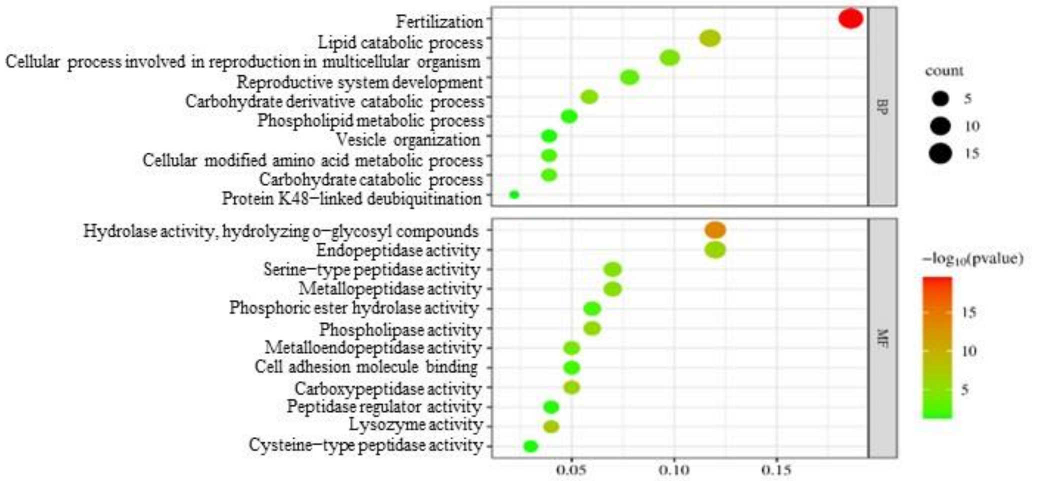

Figure S4. GO-term analysis in *Nrdp1*$^{+/-}$ and *Nrdp1*$^{-/-}$ mouse sperm. (A) Biological process and molecular function enrichment of upregulated proteins (374) in *Nrdp1*$^{-/-}$ sperm. BP: biological process; MF: molecular function, colored by *p*-values. (B) Biological process and molecular function enrichment analyses of downregulated proteins (113) in *Nrdp1*$^{-/-}$ sperm. BP: biological process; MF: molecular function, colored by *p*-values.
